# High Mobility Group Box 1: Biological Functions and Relevance in Oxidative Stress Related Chronic Diseases

**DOI:** 10.3390/cells11050849

**Published:** 2022-03-01

**Authors:** Simona Taverna, Alessandro Tonacci, Maria Ferraro, Giuseppe Cammarata, Giuseppina Cuttitta, Salvatore Bucchieri, Elisabetta Pace, Sebastiano Gangemi

**Affiliations:** 1 Institute of Translational Pharmacology (IFT), National Research Council of Italy (CNR), 90146 Palermo, Italy; simona.taverna@cnr.it (S.T.); maria.ferraro@cnr.it (M.F.); giuseppe.cammarata@cnr.it (G.C.); giuseppina.cuttitta@cnr.it (G.C.); salvatore.bucchieri@cnr.it (S.B.); 2 Institute for Biomedical Research and Innovation (IRIB), National Research Council of Italy (CNR), 90146 Palermo, Italy; 3 Clinical Physiology Institute (IFC), National Research Council of Italy (CNR), 56124 Pisa, Italy; atonacci@ifc.cnr.it; 4 Department of Clinical and Experimental Medicine, School and Division of Allergy and Clinical Immunology, Azienda Ospedaliera Universitaria Policlinico “G. Martino”, University of Messina, 98125 Messina, Italy; gangemis@unime.it

**Keywords:** high-mobility group box 1, damage-associated molecular pattern molecules, inflammation, oxidative stress related chronic diseases, cancer, diabetes, respiratory diseases, cardiovascular diseases, neurological diseases

## Abstract

In the early 1970s, a group of non-histone nuclear proteins with high electrophoretic mobility was discovered and named high-mobility group (HMG) proteins. High-mobility group box 1 (HMGB1) is the most studied HMG protein that detects and coordinates cellular stress response. The biological function of HMGB1 depends on its subcellular localization and expression. It plays a critical role in the nucleus and cytoplasm as DNA chaperone, chromosome gatekeeper, autophagy maintainer, and protector from apoptotic cell death. HMGB1 also functions as an extracellular alarmin acting as a damage-associated molecular pattern molecule (DAMP). Recent findings describe HMGB1 as a sophisticated signal of danger, with a pleiotropic function, which is useful as a clinical biomarker for several disorders. HMGB1 has emerged as a mediator in acute and chronic inflammation. Furthermore, HMGB1 targeting can induce beneficial effects on oxidative stress related diseases. This review focus on HMGB1 redox status, localization, mechanisms of release, binding with receptors, and its activities in different oxidative stress-related chronic diseases. Since a growing number of reports show the key role of HMGB1 in socially relevant pathological conditions, to our knowledge, for the first time, here we analyze the scientific literature, evaluating the number of publications focusing on HMGB1 in humans and animal models, per year, from 2006 to 2021 and the number of records published, yearly, per disease and category (studies on humans and animal models).

## 1. Introduction

The cells are challenged by exogenous stimuli that may cause injury or death. The cellular alarm system actives endogenous molecules called alarmins or damage-associated molecular pattern molecules (DAMPs) to signal danger-induced cellular stress [1,2]. DAMPs have important intracellular effects in homeostasis; they promote inflammation when released in extracellular space in response to danger signals.

High-mobility group box-1 (HMGB1) protein, also called amphoterin or HMG, is considered an alarmin, discovered in calf thymus and named according to its high electrophoretic mobility [3]. HMGB1 plays a key role in acute and chronic inflammation, with effects in several diseases. It is a promising pharmacological target in various pathologies. This review offers a wide overview of HMGB1, describing (a) its redox forms, its localization and release mechanisms; (b) HMGB1 receptors; (c) its activities and effects in different pathological contexts; (d) HMGB1 modulators as new therapeutic strategies. Considering the growing number of reports showing the pleiotropic role of HMGB1, the novelty of this review is related to the methodology employed for the analysis of the scientific literature evaluating the number of publications focusing on HMGB1 in humans and animal models per year. We have analyzed scientific literature from 2006 to 2021 and evaluated the number of records published, yearly, per disease and category (studies on humans and animal models). Since HMGB1 has been implicated in the pathogenesis of several oxidative stress-related chronic diseases, and considering that the topic is very broad, we discuss the role of HMGB1 in the principal socially relevant chronic diseases (cancer, diabetes, neural, cardiovascular, and respiratory disorders) with the largest number of papers published from 2006 to 2021.

### 1.1. HMGB1 Structure

The high mobility group (HMG) superfamily was discovered for the first time in 1973 [4] and includes three families: HMGA, HMGB, and HMGN. The HMGB family has four components (HMGB1, HMGB2, HMGB3, and HMGB4), is highly conserved evolutionarily, and has the most abundant proteins among HMGs [5].

**Figure 1:**
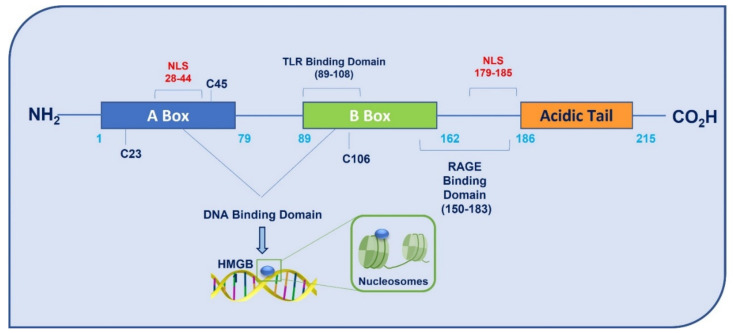
Schematic representation of HMGB1 structure. HMGB1 has 215 amino acids organized in two proximal homologous DNA binding domains, A-box, B-box, and a C-terminal acidic tail with repeated acid residues. HMGB1 has two nuclear localization signals (NLS1 and NLS2), one TLR-binding domain and one RAGE-binding domain.

HMGB1, encoded by a gene located on chromosome 13q12, is a structural non-histone chromatin-binding protein, with 215 amino acids and a molecular weight of 25-kDa. The protein contains two proximal homologous DNA binding domains, called A–box (9–79 aa) and B–box (95–163 aa), and a C–terminal acidic tail (186–215 aa) with repeated residues of glutamic and aspartic acid. Two nuclear localization signals (NLS1 and NLS2) and two nuclear export signals (NESs) drive the translocation of HMGB1 from nucleus to cytoplasm [6] (Figure 1).

**Figure 2:**
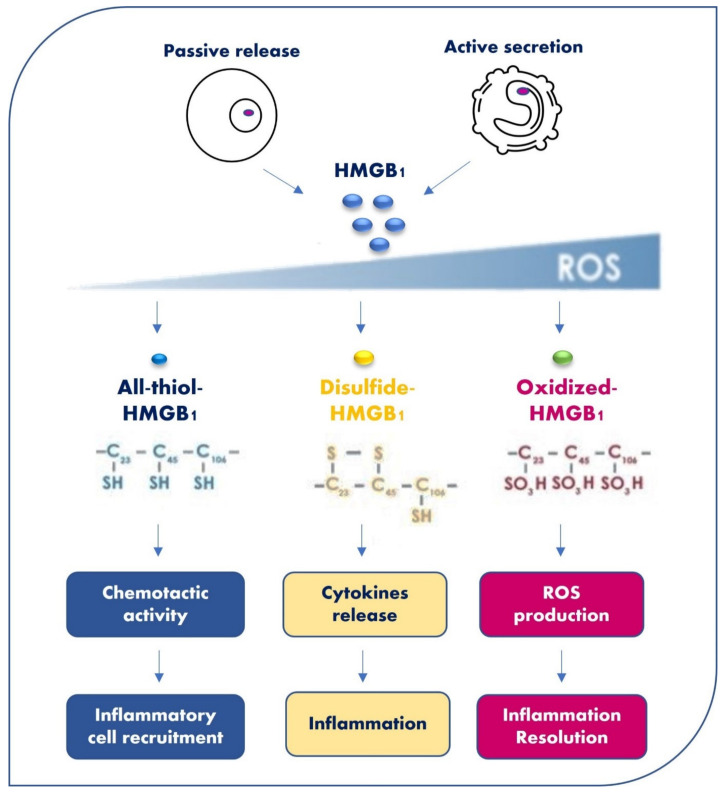
Three HMGB1 redox forms and their functions. HMGB1 has three conserved cysteines (C) in positions 23, 45, and 106. C23 and C45 can form an intermolecular disulfide bond, and C106 remains in reduced thiol state. The three redox forms of HMGB1, all-thiol-HMGB1, disulfide-HMGB1, and oxidized HMGB1 are generated by the different status of each C. HMGB1 in each redox status has a different function.

HMGB1 has three conserved cysteines (C) in positions 23, 45 (A-Box), and 106 (B-Box). C23 and C45 can form intermolecular disulfide bonds, and C106 remains in a reduced thiol state. The three redox forms of HMGB1 are all-thiol-HMGB1, disulfide-HMGB1, and oxidized HMGB1, which are generated by the different status of each C [6]. All-thiol-HMGB1 is prevalent in the nucleus and has all three cysteines reduced (Figure 2). This form creates a heterocomplex with C-X-C motif chemokine 12 (CXCL-12), which enhances its chemotactic activity for monocytes via chemokine C-X-C receptor 4 (CXCR-4), thereby contributing to inflammatory cells’ recruitment [7]. These immune cells produce the disulfide HMGB1 by oxidizing extracellular HMGB1 via reactive oxygen species (ROS) formation [8]. The disulfide-HMGB1 is produced a few hours after all-thiol-HMGB1. It activates monocytes and macrophages to release inflammatory mediators as cytokines and chemokines by binding to Toll-like receptors (TLR) 2 and 4 [9]. This binding induces the translocation of the nuclear factor kappa-light-chain-enhancer of activated B cells (NF-κB) to nucleus and pro-inflammatory cytokines transcription, as tumor necrosis factor (TNF-a), interleukins (ILs) as IL-1, IL-6, and IL-8. The oxidized HMGB1, induced by constant ROS production, acts with a feedback mechanism when inflammation resolves [6] (Figure 2).

### 1.2. HMGB1 Localization

**Figure 3:**
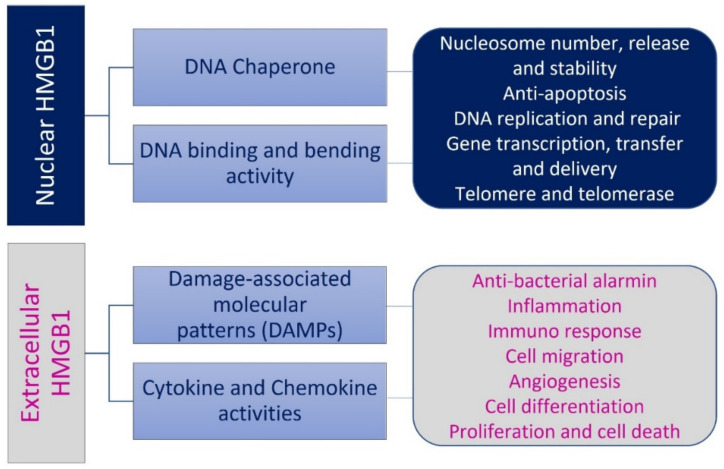
The different roles of HMGB1 depend on its localization. Nuclear HMGB1 DNA acts as a chaperone and it has activities of DNA binding and bending. Extracellular HMGB1 activates cytokine and chemokine pathways and functions as damage-associated molecular patterns (DAMPs).

The localization of HMGB1 is crucial to function and its multiple roles depend on tissue, the production of cytotypes, and on different stress signals (Figure 3). HMGB1 is localized in nucleus, cytosol, or extracellular space where it is passively released by lytic cell death or actively secreted by viable cells. When HMGB1 shuttles from nucleus to cytoplasm or it is released, it modulates cell stress responses and inflammation [3]. In physiological conditions, HMGB1 is anchored into the nucleus, and it is more concentrated in the nuclear compartment than in the cytoplasm. HMGB1 binds chromatin in the nucleus and, after damage signals, moves to the cytosol. The nucleus–cytoplasm HMGB1 ratio is about 30:1. In the nucleus, HMGB1 acts as a molecular chaperone by binding to double stranded DNA in a non-specific manner through the A and B boxes. These domains are responsible for the stabilization and maintenance of the nucleosome structure, regulation of gene transcription, chromatin condensation, and DNA replication, recombination, and repair [9]. HMGB1 is the most mobile protein that shuttles from nucleus to cytosol within 1–2 s. Considering its mobility, HMGB1, when NLS is modified, localizes in mitochondria, lysosomes, and plasma membranes [6].

### 1.3. HMGB1 Release Mechanisms

HMGB1 is expressed in different tissues and high levels have been detected in the spleen and thymus. HMGB1 is released by several cytotypes, such as macrophages, monocytes, and endothelial cells. Nuclear HMGB1 is passively released, in non-acetylated and fully reduced form, into extracellular space by lytic cell death after cell membrane damage [10].

HMGB1 can undergo several post-translational modifications, such as glycosylation, methylation, ADP-ribosylation, acetylation, phosphorylation, and oxidation. The phosphorylation of serine residues of NLSs, catalysed by different protein kinases, decreases HMGB1 DNA binding/bending activity and influences HMGB1 nuclear/cytoplasmic distribution and its release. The hyperacetylation of HMGB1 avoids the constant bidirectional transfer of HMGB1 from cytoplasm to nucleus and leads to cytoplasmic accumulation of HMGB1. Nuclear hyperacetylation is also achieved through the induction of histone-acetylase (HAT) activity and the decrease of histone-deacetylase (HDAC) activity. In this regard, it has been previously demonstrated that oxidative stress due to cigarette smoke exposure reduces HDAC2, HDAC3, and sirtuin 1 (SIRT1) expression/activity [11,12,13]. Agents such as metformin, resveratrol, and curcumin, enhancing SIRT1 deacetylase activity, lead to the decrease of extracellular HMGB1 release and the inhibition of HMGB1-dependent inflammation [14]. Depending on the nature of cellular insults and cytotypes, the release mechanisms of HMGB1 are controlled at various levels, including activation of transcription factors such as NF-κB and the nucleotide-binding oligomerization domain (NOD)-like receptor family pyrin domain containing protein three (NLRP3) inflammasome. The release of HMGB1 induces intracellular ROS formation, originating from mitochondrial or generated by NOX family enzymes activation [12]. ROS production induces calcium (Ca) overload, which in turn actives Ca-dependent enzymes, protein kinase C (PKC), and Ca-calmodulin-dependent protein kinase IV (CaMKIV). These enzymes are involved in post-translational modifications, as phosphorylation of nuclear HMGB1 [13].

Although the effects of extracellular HMGB1 are well-known, the mechanism of programmed and active release has not been thoroughly investigated [15]. Recently, it was proposed that the regulated secretion of HMGB1 implies its packaging into intracellular vesicles, such as lysosomes or autophagosomes. These vesicles can release HMGB1 after their fusion with the plasma membrane, but the signal controlling HMGB1 packaging into the vesicles and how HMGB1 resists proteolysis into lysosomes remain unclear [15].

Recently, HMGB1 has been described in extracellular vesicles (EVs) [16,17]. EVs are nanoscale lipidic particles released from all cytotypes, in physiological and pathological conditions, shuttling proteins, lipids, and nucleic acids [18,19,20]. EVs can be collected by several biofluids, such as serum, plasma, saliva, nasal secretions, urine, and malignant effusions [21,22]. EVs play a pleotropic role in health and disease states, and they have a key role in intercellular communication and immune system modulation [23,24]. In vivo and in vitro studies have demonstrated that endothelial cells (ECs) and endothelial-vascular smooth muscle cells (VSMC) release EVs containing HMGB1 to regulate vascular homoeostasis. EVs released by ECs induce a senescent, pro-inflammatory, and hypertrophic VSMC phenotype with a mechanism involving HMGB proteins [25]. HMGB1 contained in EVs released by gastric cancer cells induces autophagy and pro-tumor activation of neutrophils via the HMGB1/TLR4/NF-κB axis [26]. Furthermore, EVs containing HMGB1 released by bone marrow mesenchymal stem cells (BMSC) mitigate the damage caused by smoke inhalation. Smoke can induce the expression of HMGB1, NF-κB, and inflammatory and apoptotic factors. BMSC-EVs significantly inhibited apoptotic factors, including cleaved caspase-3, Bax, and c-Jun, and reversed HMGB1 increase. These findings suggest that EVs could be used as a potential cell-free therapeutic strategy for inhalation lung injury [27]. HMGB1 release is also induced by inflammasome activation, via gasdermin D, a pore-forming protein that causes cellular lysis [15].

The evaluation of released HMGB1 can be useful for monitoring the progression of oxidative chronic diseases. Accurate measurement of HMGB1 in plasma/serum samples is not easy; its concentration in the bloodstream is caused by a balanced process of release by activated immune cells or necrotic cells and a controlled neutralization or elimination [28]. Several methods have been proposed to measure HMGB1 concentrations in biological fluids, including homemade immunoblots and a commercial enzyme-linked immunosorbent assay (ELISA) using specific anti-HMGB1 antibodies. ELISA is a common and practical method to quantify extracellular HMGB1, but different molecules are complexed with HMGB1 and may interfere with its detection by this assay [29]. In order to overcome this limit, the perchloric acid-ELISA (PCA-ELISA), a technical improvement of classical ELISA, which allows the dissociation of HMGB1 containing complexes was proposed. In PCA-ELISA, the samples were treated with PCA before ELISA to efficiently detect the masked form of HMGB1 [30]. Despite the advances in ELISA, which remains the principal method for total HMGB1 measurement, there is a lack of antibodies with the specificity to measure the isoforms resulting from post-translation modifications such as acetylation and oxidation, which determine the location and function of HMGB1.

Another precise method for HMGB1 quantification is the electromobility shift assay (EMSA). This sensitive and specific technique allows the detection of very high affinity complexes formed between HMGB1 and radiolabelled hemicatenaned DNA loops [31].

Recently, an antibody-free sample preparation method followed by liquid chromatography joined with tandem mass spectrometry (LC-MS/MS) to measure HMGB1 in human plasma has been developed. The reversed-phase liquid chromatography separation of intact HMGB1 in diluted plasma produced a higher degree of HMGB1 purification than immunoaffinity extraction, which is useful for overcoming its low recovery in body fluids [32]. Currently, the analytical methods represent a compromise between isoform resolution (LC/MS) and ease of use with high throughput measurement of total HMGB1 (ELISA) with the possibility of reducing interference (PCA-ELISA).

### 1.4. HMGB1 Receptors

HMGB1 binds several extracellular receptors, such as receptors for advanced glycation end products (RAGE), TLR9, TLR4, TLR2 integrins, α-synuclein filaments, proteoglycans, T-cell immunoglobulin, and mucin domain (TIM-3), triggering receptors expressed on myeloid cells-1 (TREM1), CXCR4, N-methyl-D-aspartate receptor (NMDAR), and cluster of differentiation 24 (CD24). Each domain of HMGB1 interacts with specific receptors; specifically, the residues 150–183 are responsible for binding to RAGE. The residues 89–108 bind TLR4, and the domain 7–74 bind p53 [33].

Although several HMGB1 receptors are known, only two receptor systems, RAGE and TLR-4, have been fully elucidated as HMGB1 receptors [34].

RAGE, a member of the immunoglobulin superfamily, is a transmembrane receptor with an extracellular domain, a transmembrane domain, and a cytoplasmic tail of 43-amino acids. The extracellular domain is responsible for ligand binding, and the cytoplasmic tail is involved in the intracellular signaling transduction. RAGE was previously considered a receptor for advanced glycation end products (AGEs), but is now recognized as a receptor multi-ligand, including HMGB1. It was demonstrated that RAGE is involved in HMGB1-induced cell inflammation, proliferation, migration, and immunity. Moreover, extracellular HMGB1 stimulates RAGE expression in different cytotypes. The HMGB1-RAGE axis represents a potential target for oxidative stress-related chronic diseases [35].

TLRs are a type I transmembrane superfamily with extracellular leucine-rich repeat (LRR) domains and a cytoplasmic Toll/IL-1 receptor (TIR) domain. TLRs recognize several danger signals, including DAMPs, activating an innate immunity response. The two major signal transduction pathways are involved in TLR activation. MyD88-dependent pathway is important for the production of inflammatory cytokines, whereas MyD88-independent pathway is required for type I IFN production and DCs maturation. HMGB1 interacts with different TLRs, such as TLR2, TLR4, and TLR9, to activate the NF-kB pathway to induce cytokine and chemokine expression [35].

Many receptors, such as CXCR4, proteoglycans, integrins, TIM-3, and CD24, bind to HMGB1 through multi-protein complex formation [34].

CXCR4 is a member of G protein-coupled receptors, and its major ligand is CXCL12. After binding to CXCL12, HMGB1 forms a heterocomplex and binds to CXCR4, inducing the recruitment of inflammatory cells to injured tissues [36].

The proteoglycans are glycosylated proteins that covalently bond to anionic glycosaminoglycan. The binding of HMGB1 to heparan sulfate is mediated by RAGE.

The integrins are adhesion proteins that play critical roles in leukocyte recruitment, in particular Mac-1, a leukocyte integrin involved in inflammatory cell recruitment that can regulate HMGB1 activity [37].

CD24 is a cell surface GPI-anchored mucin-like glycoprotein expressed by different cytotypes as neuronal, hematopoietic, and epithelial cells. CD24 functions as a negative DAMP regulator, and CD24 inhibits HMGB1-induced NF-kB activation and the pro-inflammatory cytokine production. CD24 and TIM-3 are considered negative receptors, since they inhibit the immune responses induced by HMGB1 in tumor-associated dendritic cells and macrophages, respectively [38].

The danger signals activate innate and adaptive immunity and promote tissue repair [39]. HMGB1 binds with high affinity to different receptors, mediating the immune response to necrosis and immune cell invasion to pathogens, sepsis, and trauma [40]. Extracellular HMGB1 alarms the innate immunity, recruiting different cytotypes as mesangioblasts, inflammatory, smooth muscle, and stem cells. Under stress conditions, HMGB1 can sustain long-term inflammation. Furthermore, HMGB1 functions as an immune adjuvant inducing a strong response to activate or suppress T cells, dendritic cells (DC), and endothelial cells. As mentioned above, activated immune cells (monocytes, macrophages, DCs) and endothelial cells release HMGB1, which creates a positive feedback loop inducing the secretion of cytokines and chemokines via different pathways [41].

### 1.5. HMGB1 Activities

The levels of HMGB1 correlate with the differentiation stages of immune cells [42]. HMGB1 mediates innate and adaptive inflammatory responses to injury. In physiological conditions, this alarmin is inside the nucleus of quiescent macrophages/monocytes. In response to exogenous and endogenous inflammatory stimuli, HMGB1 can be secreted actively by macrophages/monocytes [43]. HMGB1 activates macrophages and DCs to release TNF-α and produce inflammatory cytokine and chemokine via the TLR4/MD2/MyD88/NFκB pathway. Activated macrophages not only secrete HMGB1 in extracellular space, but also express receptors (RAGE, TLR-2/4) on their membrane to which HMGB1 binds [44]. HMGB1 stimulates, via RAGE, inflammasome and caspase-1 in macrophages, leading to pyroptosis [44,45]. The internalization of RAGE/HMGB1, in macrophages, leads to lysosomes’ rupturing and releasing cathepsins into the cytosol, thereby contributing to inflammasome activation. Since ECs of vasculature form an important barrier preventing inflammatory processes [3], the loss of endothelial cell monolayer integrity can exacerbate organ damage. HMGB1 binding to ECs can upregulate, in a dose dependent manner, the expression of adhesion molecules such as ICAM-1, VCAM-1, and E-selectin, which can increase adhesion and movement of inflammatory cells across the vascular endothelium [46]. In response to HMGB1, ECs increase the production of proinflammatory IL-8 and G-CSF. HMGB1 also plays an important role in regulating EC cytoskeletal rearrangement and vascular permeability. It was reported that high levels of HMGB1 collected by sera from patients with sepsis induce loss of vascular endothelial monolayer integrity, causing the formation of F-actin stress fibers and VE-cadherin delocalization. HMGB1 alters endothelial integrity and trans-endothelial electrical resistance in a dose and time-dependent manner. These effects are mediated by RAGE and p38 mitogen-activated protein kinase (MAPK) signaling [47]. Moreover, the neutrophils are the main actors in bacterial clearance in sepsis and have been implicated in organ damage in sepsis and trauma. HMGB1, acting via RAGE-dependent signaling, decreases NADPH oxidase activity in neutrophils [48].

HMGB1 plays a central role in autophagy induction. Depending on different localizations, HMGB1 contributes in several ways to autophagy activation. In nucleus, HMGB1 upregulates HSP27 expression to induce autophagy. In cytoplasm, the Beclin-1/PI3K-III complex is activated by HMGB1, thereby promoting autophagy. Extracellular HMGB1, released by cancer cells, binding RAGE can induce autophagy activity in nearby cells [49].

Epigenetic mechanisms, such as the activities of microRNAs (miRNAs), can regulate HMGB1 expression. This alarmin can also modulate the expression of several miRNAs involved in oxidative stress-related chronic diseases. In different cells or tissues, the expression profile of the miRNAs is modified following DAMP stimulation [50].

MiRNAs are a family of small, noncoding RNAs of 18–22 nucleotides that regulate gene expression. They play an important role in several biological processes, such as the maintenance of immune homeostasis, cancer progression, and inflammation [51].

It was reported that HMGB1 induces the expression of miR-206, which inhibits the tissue inhibitor of metalloproteinase 3 (TIMP3), a physiological regulator of cardiac regeneration [52]. The activation of the HMGB1:RAGE axis increases miR-221 and miR-222 expression in thyroid cancer cells, thereby facilitating tumor progression [53].

Moreover, miRNAs bind to complementary target mRNAs, leading to mRNA degradation or translational inhibition. Recently, it was demonstrated that several miRNAs modulate HMGB1 expression and its functions [54]. The epigenetic modulation by miRNAs of the HMGB1 gene is involved in pulmonary disorders. MiR-627 targeting HMGB1 modulates TGF-β, which induces pulmonary fibrosis via the NF-κB regulatory loop [55]. It was also demonstrated that miR-574-5p attenuates acute respiratory distress syndrome by targeting HMGB1. MiR-574-5p is a negative regulator of inflammatory responses by controlling critical aspects of respiratory system homeostasis [56]. Moreover, miRNAs can affect HMGB1 gene expression, modulating cancer progression. HMGB1 is a direct target of miR-320a, and the expression level of miR-320a might regulate the invasion and metastasis of hepatocellular carcinoma (HCC) cells by targeting the HMGB1 pathway [57]. Recently, it was also reported that in human cervical cancer cells, miR-142-3p targets HMGB1, inducing apoptosis and inhibiting proliferation, migration, and invasion of cancer cells [58].

## 2. Search Strategy

The search was conducted in PubMed and limited to articles published between 1 January 2006 and 31 October 2021. Original articles were included in the evaluation, whereas review articles, conference papers, and articles outside the scope of the review were excluded. Only articles published in English were considered. Relevant articles were searched as follows: (“Hmgb-1” OR “high-mobility group protein 1” AND “*”), where “*” stands for: (i) “cardiovascular diseases” OR “cardiovascular” OR “cardiac”; (ii) “cancer” OR “tumor” OR “tumour” OR “oncology” OR “neoplasm”; (iii) “neurological disease” OR “neurological disorder” OR “neurodegenerative disorder”; (iv) “respiratory disease” OR “respiratory disorder” OR “lung disorder”; (v) “diabetes” OR “metabolic disorder” Articles were then sorted by relevance and screened for coherence with the scope of the review.

## 3. Results and Discussion

### 3.1. Studies on Human Subjects

**Figure 4:**
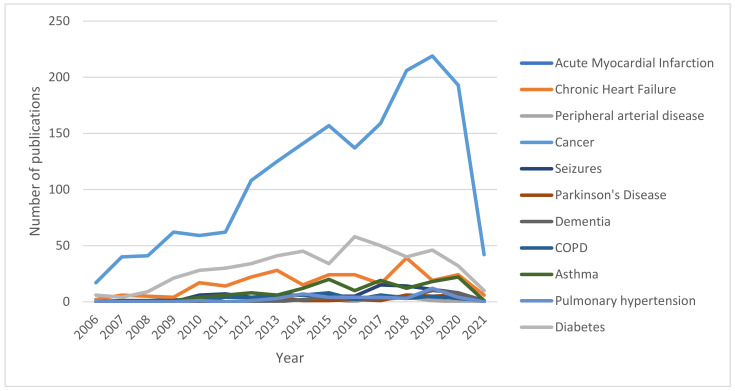
Overall number of publications involving HMGB1 among humans and other animal models per year.

**Figure 5:**
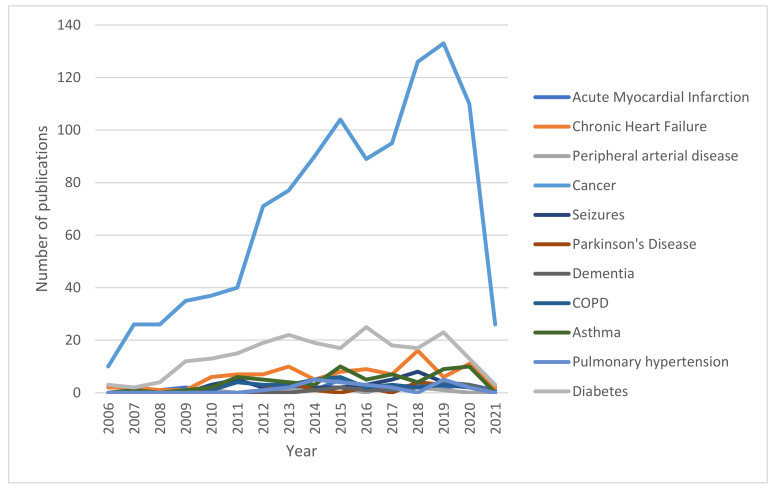
Number of records published yearly between 2006 and 2021 per disease (studies on human subjects).

Studies investigating HMGB1 in the most prevalent pathological categories among human subjects represent the relative majority in the scientific literature (Figure 4). As displayed in Table 1 and visually reported in Figure 5, cancer represents the condition that is the most widely investigated, with more than 1000 records retrieved from PubMed in the last 15 years. The recent related trend is, overall, increasing, with the peak having been reached in 2019, with 133 records found. On the other hand, diabetes is widely investigated, with 225 records retrieved, and shows a similar trend to cancer, with the highest number of publications found in 2016, with 25 articles. Cardiovascular (mainly concerning stroke), respiratory (mostly, asthma), and, lastly, neurological conditions followed, with roughly 100 articles published in the last 15 years per category. All three categories peaked, in terms of the highest number of publications, between 2015 and 2018, and, concerning cardiovascular and neurological diseases, there seems to be a somewhat decreasing interest in the final years considered in the present analysis.

**Figure 6:**
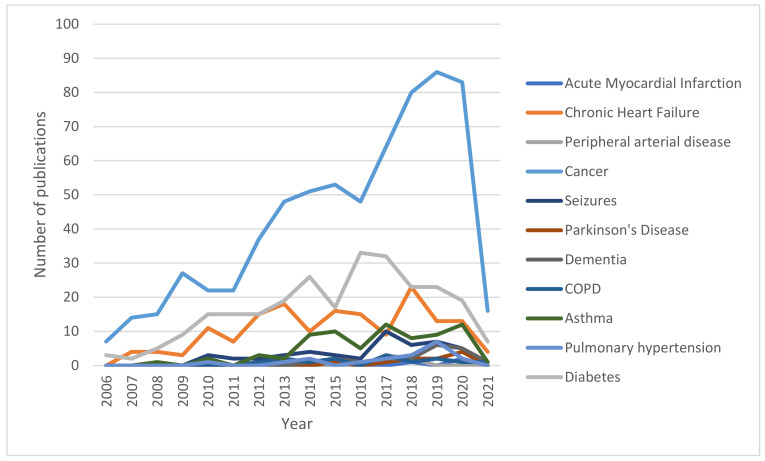
Number of records published yearly between 2006 and 2021 per disease (studies on animal models).

When it comes to the analysis of studies conducted on other animal models, the prevalence of cancer over diabetes and cardiovascular, respiratory, and neurological diseases remain. However, the yearly trends reported in Table 2 and displayed in Figure 6 suggest a decreasing interest in animal models’ studies dealing with diabetes and cardiovascular diseases in the last years, counterbalanced by an increasing number of records about cancer and respiratory and neurological diseases, possibly suggesting a different level of clinical importance related to the retrievals of both animal and human studies on different domains of the healthcare panorama.

### 3.2. Role of HMGB1 in Cancer

HMGB1 expression is upregulated in cancer and downregulated during aging, suggesting a critical role in development and tumor progression. The alterations of HMGB1 locations and the modulation of its expression levels are associated with cancer hallmarks described by Hanahan and Weinberg [59]. In human cancers such as colon, breast, lung, cervical, and liver carcinomas, an aberrant release of HMGB1 has been shown [60]. HMGB1 has a dual role in cancer development with pro- and anti-tumoral effects [61]. In tumor progression, HMGB1 can facilitate both cell survival and death by modulating various signaling pathways [6]. Neoplastic cells secrete HMGB1 under hypoxia, sustaining the inflammatory tumor microenvironment. Extracellular HMGB1, in turn, activates the NF-κB and inflammasome pathways to induce proinflammatory cytokine release. This loop enhances inflammatory responses, tumor formation, and metastasis [60]. RAGE and TLR4 are involved in the inflammatory response, mediated by HMGB1, during tumorigenesis. HMGB1 is also involved in the energy metabolism of cancer. Extracellular HMGB1 induces mitochondrial RAGE expression and translocation, which in turn enhances ATP production and mitochondrial activity. HMGB1 promotes malignant phenotypes of cancer cells, increasing epithelial mesenchymal transition (EMT), invasion, angiogenesis, and metastasis. Since hypoxia causes HMGB1 release and increased RAGE expression in the tumor microenvironment, the upregulated HMGB1/RAGE complex, activating the NF-κB pathway, induces the expression of proangiogenic growth factors such as the vascular endothelial growth factor (VEGF) and their receptors. It was reported that HMGB1 released by cholangiocarcinoma cells promotes angiogenesis and increased VEGFR2 in endothelial cells. High expression of HMGB1 in blood and tissues is associated with an increase of microvascular density and poor prognosis. The analysis of postoperative serum of cholangiocarcinoma patients indicates that HMGB1 could predict high recurrence and adverse prognosis for cholangiocarcinoma [62]. HMGB1 also promotes angiogenesis by regulating hypoxia-inducible factor 1 (HIF-1α) expression via the PI3K/AKT pathway in breast cancer [63]. Cancer immune surveillance is considered an important process to inhibit carcinogenesis and maintain cellular homeostasis. HMGB1 has immunosuppressive and immune stimulatory activities, depending on redox state, receptors, and targeted cells. This alarmin can contribute to the inhibition of antitumor immunity, inducing apoptosis in macrophage-derived dendritic cells, which reduce host anti-cancer immunity [6]. The overexpression of HMGB1 has been associated with a poor survival rate in various cancers. HMGB1 can represent an oncogene-like biomarker of lung cancer [64]. In the NSCLC tissue, HMGB1 expression is increased with respect to normal tissue, and it has been associated with low patient survival. It was reported that miR-200c targeting the HMGB1 gene and reducing its expression attenuated EMT, invasion, and migration of NSCLC [65].

Conversely, HMGB1 can exhibit anti-tumor effects, interacting with tumor suppressor factors, increasing genome instability and autophagy. In breast cancer, intracellular HMGB1 binds the retinoblastoma (RB) protein, a well-known tumor suppressor factor, inhibiting cancer progression [41,66]. In pancreatic cancer, intracellular HMGB1 is considered a novel tumor suppressor that sustains chromosome stability and limits pro-inflammatory nucleosome activity and release [67,68].

Our analysis indicates that in the last 15 years, about 1000 records focused on HMGB1 and cancers in humans have been published (Figure 4 and Table 1). The data reveal an increasing trend of papers in this field, with the peak of publications having been reached in 2019, with 133 records found. The data also show that studies on HMGB1 and cancers conducted on animal models have a high prevalence and confirm the increasing trend of publication.

**Figure 7:**
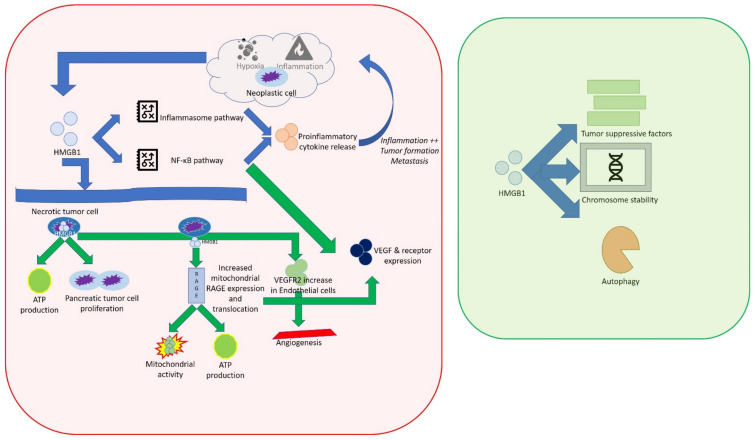
Schematic representation of the effects of HMGB1 in cancer.

Overall, HMGB1, as a function of its redox state and its localization, can be considered a potential risk factor, a new biomarker, or a therapeutic target in cancer management (Figure 7).

### 3.3. Role of HMGB1 in Neural System

In the neural system, HMGB1 has a key role in inflammatory response and it is involved in several pathological conditions such as ischemic stroke, septic shock, epilepsy, traumatic brain injury, Alzheimer’s, Parkinson’s disease, and multiple sclerosis [69].

In cerebral ischemia, during the acute phase, DAMPs are massively released and mediate signaling for proinflammatory activation of microglia, astrocytes, and ECs. DAMPs also cause neutrophil infiltration into damaged brain tissue [70]. Neutrophils are the first group of cells infiltrating the damaged brain tissue, where they produce neutrophil extracellular traps (NET). Activated neutrophils produce ROS and proinflammatory cytokines and can induce NETosis, a form of cell death, in brain parenchyma and blood vessels, exacerbating inflammation and injury following ischemia [71]. Since it was reported that all thiol and disulfide HMGB1 induce NETosis via CXCR4 and TLR4, HMGB1 may be considered a mediator of stroke [72].

After neuronal injury, significant amounts of HMGB1 from the nucleus are passively released into extracellular space. The HMGB1/TLR4 axis is a key initiator of neuroinflammation; HMGB1 is actively released by neurons and glia cells after inflammasome stimulation and TLR4 and RAGE activation on target cells. MyD88, a downstream effector molecule of TLR signaling, is involved in HMGB1-mediated post-ischemic inflammatory response and enhances stroke lesions when compared to MyD88 knockout mice [73].

In the blood of patients with ischemic stroke, higher levels of HMGB1 compared to age and sex-matched controls have been detected [74]. A strong correlation between matrix metalloproteinase 9 (MMP-9) and HMGB1 levels, which was associated with poor outcomes, has been reported in ischemic stroke patients [75]. Various studies have demonstrated that blocking or modulating HMGB1 by compounds such as statins or by RNA interference provide neuroprotective effects against ischemic stroke [76,77]. Recently, HMGB1 has been studied for its role in epilepsy. It might be involved in epileptogenesis through blood–brain barrier (BBB) disruption and induction of inflammatory processes. HMGB1 can also cause disruption of BBB, leading to cognitive decline in aged rats [74].

Moreover, HMGB1 is involved in pathogenesis of the two most common neurological disorders: Alzheimer’s (AD) and Parkinson’s disease (PD). Since neuroinflammation is an important feature of AD and HMGB1 is an actor in this process, it can be considered a mediator of AD pathogenesis. High levels of HMGB1, RAGE, and TLR4 has been detected in peripheral blood and tissues of AD patients [78]. The activation of RAGE signaling in AD is involved in production and aggregation of beta amyloid (Aβ), formation of neurofibrillary tangles, disruption of synaptic transmission, and neuronal degeneration. TLR4 activation induces neuroinflammation and Aβ deposition [79]. Moreover, the activation of the HMGB1/TLR4 axis is associated with development, progression of PD, and drug treatment effectiveness. [80]. HMGB1 mediates microglial activation via NF-κB regulation. Extracellular HMGB1 accumulation in tissue or circulation contributes significantly to the pathogenesis of AD and PD [81]. The increased levels of HMGB1 in neurological diseases indicate its involvement in disease pathogenesis.

Our literature analysis indicates that about 100 articles have been published from 2006 to 2021 on HMGB1 and neurological conditions in humans. The peak of these publications was between 2015 and 2018. The data also indicate a small decreasing interest in the last years. Conversely, the studies on HMGB1 and neurological disorders conducted on animal models have a high prevalence, with an increasing trend of publications.

**Figure 8:**
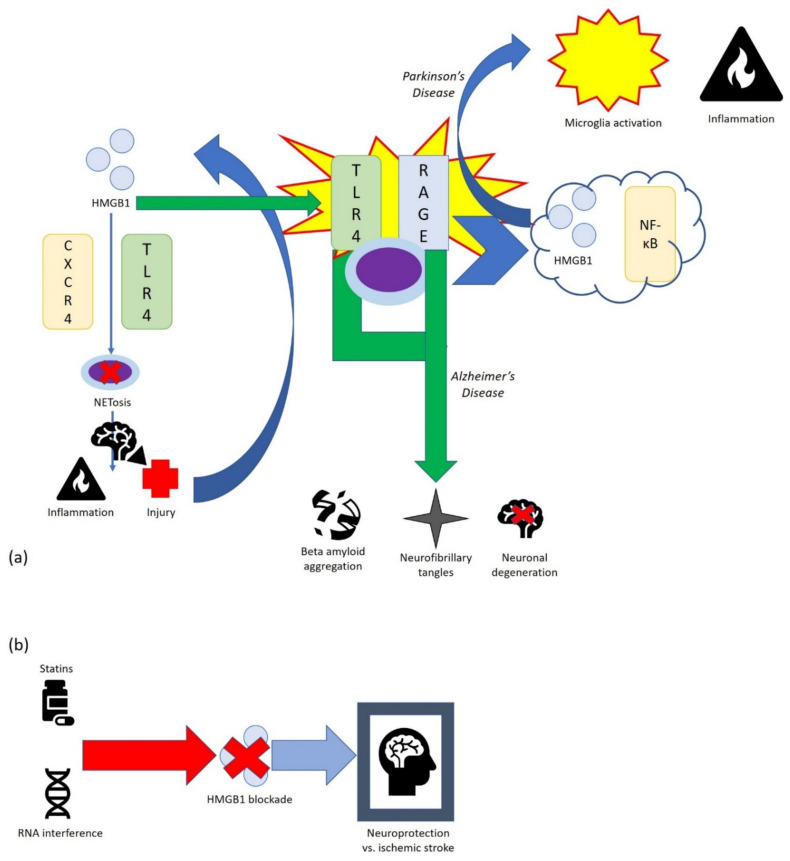
Schematic representation of the role of HMGB1 in neurological disorders (**a**) and the effect of HMGB1 blockade (**b**).

The encouraging result of HMGB1 inhibitors indicates that further research to elucidate their role in neurological diseases (Figure 8) and further clinical studies to validate their therapeutic potential are needed [82].

### 3.4. Role of HMGB1 in Cardiovascular System

Cardiovascular diseases are major causes of morbidity and mortality worldwide [83]. HMGB1 is involved in a wide range of cardiovascular pathophysiology events related to cardiac remodeling, cardiac hypertrophy, atherosclerosis, and cardiomyopathy, myocardial infarction (MI), and heart failure [34].

HMGB1 is considered a “Janus face” in heart disease, since it is involved in cardiac tissue damage and plays a role in cardiac tissue repair and regeneration [8]. HMGB1 has both pro-inflammatory and restorative effects on tissue recovery and myocardium regeneration [34].

MI causes an intense inflammatory response that is essential for cardiac repair, but it is also implicated in the pathogenesis of post-infarction remodeling and heart failure [84].

**Figure 9:**
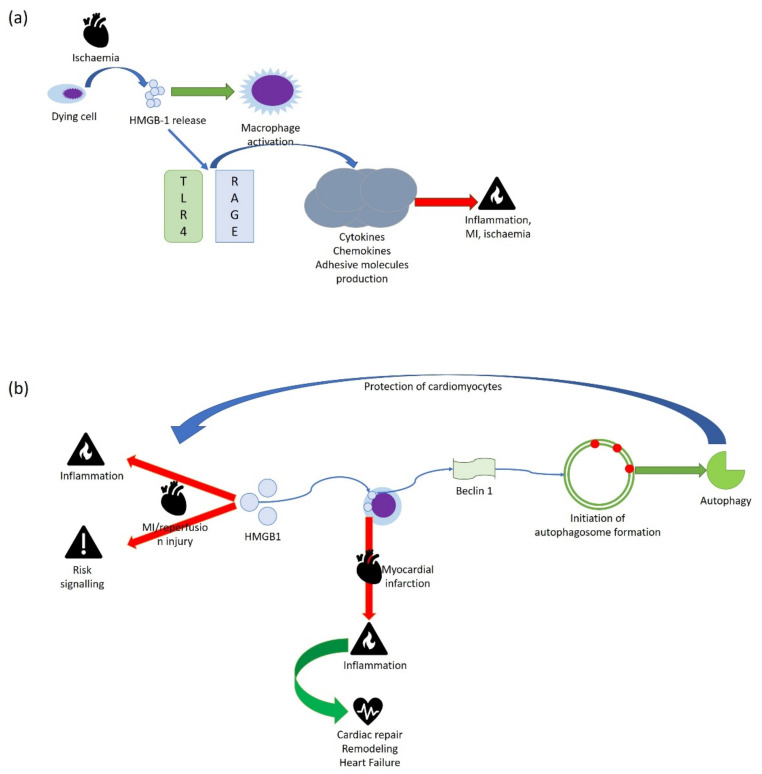
Schematic representation of the dual role of HMGB1 in cardiac disorders. Effects of HMGB1 in ischemia, MI, and inflammation (**a**) cardiac remodeling and repair (**b**).

In an injured heart, nuclear HMGB1 translocates into cytoplasm, where it acts as a DAMPs, inducing the inflammatory response. Under ischemic stress, HMGB1 is released by dying cells to activate macrophages; extracellular HMGB1 interacts with TLR-4 and RAGE and induces the production of cytokines, chemokines, and adhesive molecules, causing inflammation and MI/ischemia (Figure 9a) [85].

Non-oxidizable HMGB1 recruits inflammatory cells to injured sites, via CXCR4, increasing cardiac fibroblast migration and indorsing poor cardiac remodeling after MI [34]. The disulphide-HMGB1 that acts as a pro-inflammatory cytokine, the further oxidation of all cysteines to sulfonates, and sulfonyl HMGB provide an anti-inflammatory signal and reduces excessive inflammatory activity [86]. The interplay between fully reduced and disulfide HMGB1 isoforms is a reversible process influenced by the redox status of the microenvironment [87].

HMGB1 is also involved in heart failure. In patients with heart failure, cardiac HMGB1 translocates from nucleus to cytoplasm, regulating the inflammatory response. The decrease of nuclear HMGB1 is associated with human cardiac failure. It was observed in the model of heart failure induced by pressure overload that maintaining a correct amount of HMGB1 can reduce the risk of cardiac hypertrophy and prevent complications [34]. It was also reported that the HMGB1:RAGE axis is important for initiating and maintaining the inflammatory response in cardiomyopathy, which causes heart failure.

As mentioned above, HMGB1 has a potential role of in cardiomyocyte survival following acute MI. A mechanism of cardiac repair, HMGB1-mediated is represented by autophagy induction. Autophagy is important for healthy cardiac function and is activated to protect cardiomyocytes from myocardial I/R injury. Cytoplasmic HMGB1 participates in autophagy by binding to autophagy-related protein beclin 1 and initiating autophagosome formation [58]. HMGB1 can help the heart to restore normal function, promoting myocardial regeneration in the infarcted area after acute MI, stimulating cardiac fibroblasts to release chemokines and cytokines, and enhancing cardiac cell proliferation and differentiation (Figure 9b)

Angiogenesis is a crucial compensatory system in the myocardial wound healing response of MI [88]. HMGB1 promotes angiogenesis, and several immune cells are involved in neovascularization.

It was reported that VEGFA, released under ischemic conditions, induces the recruitment of a subset of circulating neutrophils with high expression levels of VEGFR1, CXCR4, and MMP9, to facilitate a rapid angiogenesis at hypoxic areas [89]. Moreover, circulating monocytes can induce angiogenesis, secreting growth factors and proteases, such as FGF-2, VEGF, and MMP-9. Monocytes enrolled in ischemic tissues can act as angioblasts, developing an endothelial-like phenotype after angiogenic stimulus. These cells are known as endothelial progenitor cells and by adhering to the endothelium of ischemic areas, they participate in new vessel formation [90].

HMGB1 also induces angiogenic responses directly through angiogenic cytokine secretion or indirectly by inducing proangiogenic cells such as macrophages. HMGB1 stimulates the recruitment and stimulation of macrophages, promoting angiogenesis through FGF-2, TGFβ1, and VEGF secretion [91]. In the heart, it was demonstrated that HMGB1 induces angiogenesis after injury by upregulating VEGF expression, but further studies are needed to understand how HMGB1 induces VEGF release [92].

On the other hand, exogenous HMGB1 plays a key role in cardiac regeneration. Limana and colleagues demonstrated that administering HMGB1 to the hearts of mice a few hours after MI induces cell proliferation and differentiation, thereby enhancing cardiac regeneration and improving cardiac function [93]. The same research group reported that prolonged HMGB1 administration to an infarcted heart attenuates left ventricular dilation and increases infarcted wall thickness. HMGB1 injection in a chronically injured heart improves left ventricular function and attenuates adverse cardiac remodeling, inducing cardiac tissue regeneration and extracellular matrix degradation [52]. Moreover, these authors also reported that cardiac regeneration mediated by exogenous HMGB1 plays a key role Notch signaling pathways [94].

Literature data show that about 25 articles on HMGB1 and cardiovascular disorders (mainly MI) were published in the period we selected. The peak of these publications was between 2015 and 2018, but the data indicate a small decreasing interest in the last years. The analysis of studies of HMGB1 and cardiovascular disorders conducted on animal models indicates that they have high prevalence and the yearly trends reported in Table 2 and Figure 6 confirm a small decreasing interest. The recent findings indicate that endogenous HMGB1 can be useful as a diagnostic and prognostic biomarker in human cardiac disorders [95]. Several studies have indicated that exogenous HMGB1 can have a beneficial effect on cardiac regeneration [92].

### 3.5. Role of HMGB1 in Respiratory System

HMGB1 is an important mediator of pulmonary inflammatory responses with cytokine-like effects and a key role in the pathogenesis of lung inflammatory diseases. Nowadays, the interest of the scientific community in the role of HMGB1 in lung disorders such as chronic obstructive pulmonary disease (COPD), pulmonary hypertension (PH). and asthma is growing [96,97,98].

COPD is characterized by chronic airway inflammation and lung injury, with increased numbers of macrophages, neutrophils, and lymphocytes in airways, lung parenchyma, and pulmonary vessels. In COPD patients, HMGB1 is overexpressed in lung tissue, plasma, bronchoalveolar lavage fluid (BALF), and sputum, and it is negatively correlated to the post-bronchodilator FEV1/FVC ratio of lung function [99]. Cigarette smoke (CS) is one of the main risk factors for developing several chronic diseases such as COPD, asthma, and PH [100]. Several studies indicate that CS promotes the expression, nucleus-cytoplasmic translocation, and release of HMGB1 [99]. In acute exposure to CS in mouse models, HMGB1 translocation and release in lung epithelial cells is shown to mediate lung inflammation while CS decreases lung function and improves HMGB1 levels in a chronic (60 days) CS-exposure mouse model. CS induces HMGB1 translocation from lung epithelial cells, and HMGB1 induces an inflammatory response through TLR4/MyD88-dependent signaling in an acute (3 days) CS-exposure model. Moreover, CS exposure induces caspase 1 activation via the TLR4-TRIF-caspase-8-dependent pathway [101], thus favoring HMGB1 [102]. Accordingly, COPD patients exhibit increased systemic inflammasome activation [103]. Extracellular HMGB1 also induces cytokine release through TLR4 receptors in macrophages [104,105]. A recent study indicates that CS-induced HMGB1 translocation and release contributes to NF-κB activation and migration of macrophages in the lung through autophagy induction, providing new evidence for HMGB1 as a new target in COPD management [99]. Furthermore, bronchial epithelial cells exposed to CSE as well as the airway epithelium of smokers and of COPD patients show increased expression of TLR4, which can further amplify HMGB1-mediated responses [106,107].

Asthma, another common chronic inflammatory disease of the airways, is caused by complex interactions of genetic factors and the environment. This disorder is characterized by airway hyperresponsiveness, episodic airway obstruction, and reduced lung function. Recent studies have demonstrated that HMGB1 is involved in asthma progression [97]. HMGB1 is overexpressed in the lung tissue, serum, and sputum of patients with severe asthma, but its effects on the regulatory mechanism are unclear [108]. HMGB1 regulates airway inflammation by modulating Th2 and Th17 responses in asthmatic mice models. HMGB1 can induce Th2 and Th17 through two mechanisms. HMGB1 could directly act on naive T cells, inducing Th2 and Th17 polarization via TLR2, TLR4, and RAGE-NF-κB signaling. Alternatively, HMGB1 could mediate the maturation and antigen-presenting ability of DCs, indirectly promoting Th2 and Th17 differentiation [109]. HMGB1 is also involved in pulmonary fibrotic pathogenesis, inducing lung fibroblast differentiation to myofibroblast through the NF-κB mediated TGF-β1 release.

PH is characterized by increased pulmonary vascular remodeling, pulmonary vascular resistance, and vasoconstriction, leading to progressive right ventricular failure and death. The inflammation is considered a key disease-related factor in PH. The plasma levels of inflammatory cytokines such as in IL-1β, IL-6, TNF-α, and HMGB1, were elevated in hypoxia-induced pulmonary hypertension rat models, suggesting that HMGB1/TLR4 signaling promotes hypoxia-induced PH [110].

Very recent studies have reported that HMGB1 can be considered a potential biomarker and therapeutic target for severe COVID-19. Preliminary in vitro studies indicate that exogenous HMGB1 induces the expression of SARS-CoV-2 entry receptor ACE2 in alveolar epithelial cell lines in a RAGE-dependent manner, inducing positive feedback to accelerate viral infection and host damage. The inhibition of the HMGB1-RAGE pathway by siRNA or drugs blocks ACE2 expression, thereby suppressing SARS-CoV-2 infection. HMGB1 collected from the serum of severe COVID-19 patients is elevated compared with healthy controls, indicating a potential pathological link between HMGB1 and COVID-19 [111,112,113].

Our analysis shows that about 25 articles have been published in the last 15 years on HMGB1 and respiratory disorders (mainly on asthma). The peak of these publications was between 2015 and 2018, but the data indicate a small decreasing interest in the last years. On the other hand, the literature analysis indicates that studies on HMGB1 and respiratory disorders conducted on animal models have high prevalence, with an increasing trend.

**Figure 10:**
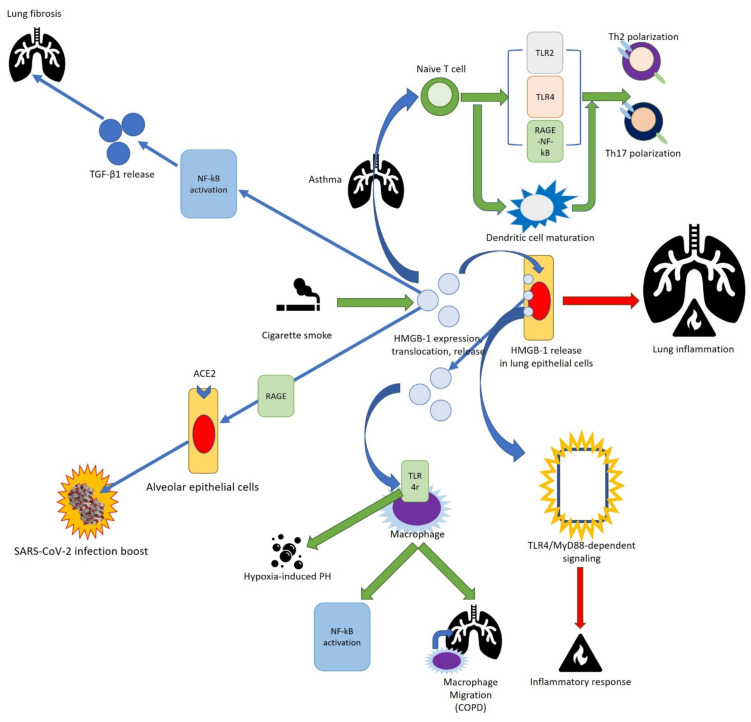
Schematic representation of the role of HMGB1 in respiratory diseases.

Although the recent findings clearly indicate HMGB1 involvement in all respiratory diseases, many studies are performed on animal models (Figure 10). In order to clarify the role HMGB1 in these pathologies, further studies on human samples are needed.

### 3.6. Role of HMGB1 in Diabetes

In recent years, diabetes has become a global challenge due to its alarming increase. Currently, despite advances in diabetes treatments, the therapies are unable to control its progression and complications. Type 1 and 2 diabetes are heterogeneous diseases, and their clinical presentation and progression can be different. The classical paradigms according to which type 2 diabetes arises only in adults and type 1 only in children seems be imprecise. In diabetes, several genetic and environmental factors can induce progressive loss of pancreatic beta-cells and their functions, which clinically leads to hyperglycaemia. Patients with hyperglycaemia in all forms of diabetes are at risk of developing the same chronic complications [114]. HMGB1 is passively released by injured pancreatic beta cells or actively secreted by DCs and macrophages that infiltrate the pancreatic islet. In type 1 diabetes, extracellular HMGB1 enhances autoimmune progression by damaging regulatory T cell stability [115]. The role of inflammation in diabetes has been widely studied and this disease is considered an inflammatory disorder. Emerging evidence shows that HMGB1 is associated with the pathogenesis of diabetes since, in diabetic patients and animal models, increased levels of HMGB1 have been observed [116]. Patients with type 2 diabetes have a state of subclinical systemic inflammation with upregulated TLR4 expression in different cytotypes and tissues. HMGB1 is overexpressed in retinas of diabetic patients and rat models with retinopathy, and circulating levels of HMGB1 are higher in type 2 diabetic patients than controls [117].

HMGB1 is involved in the onset of diabetes complications including diabetic vascular disorders such as endothelium-dependent relaxation (EDR) damage, an important element for vascular complications caused by the imbalance of vascular tone regulated by vasodilator and vasoconstrictor factors. In in vivo and in vitro models, HMGB1 may induce EDR failure through TLR-4 pathway activation [118].

Moreover, HMGB1 has a role in inflammatory responses in diabetic nephropathy; increased levels of HMGB1 are positively correlated with IL-6 and TNF-α production in the serum of diabetic patients with different degrees of renal disorders. HMGB-1/RAGE signaling activation in turn induces NF-κB, and ERK1/2 promotes diabetes induced retinal neuropathy [119].

Our analysis shows that diabetes is widely investigated, with a trend like cancer and the highest number of publications found in 2016. The papers on HMGB1 and diabetes conducted on animal models published from 2006 to 2021 have high prevalence, but the yearly trend reported in Table 2 and Figure 6 confirm a small decreasing interest in the scientific community.

These findings indicate that HMGB1 can represent a new marker to monitor diabetic progression and complication onset. Furthermore, the strategies to block the interaction between HMGB1 and its receptors may be useful in diabetes management.

### 3.7. HMGB1 Modulators as New Therapies

Since HMGB1 induces an inflammatory response, its targeting presents a promising therapeutic strategy for several diseases. HMGB1 has been studied for its therapeutic potential in sepsis, inflammation, oxidative stress-related diseases, and cancer [120].

Different strategies, including antagonist, polyclonal, and monoclonal antibodies, recombinant HMGB1, and epigenetic modulation have been shown to be successful in inhibiting HMGB1 related inflammatory processes. Many HMGB1 antagonists can block TLR4/HMGB1 or RAGE/HMGB1 pathways. The HMGB1 neutralizing antibody and recombinant HMGB1 box A protein have had positive effects in a wide range of preclinical models of inflammatory diseases [121].

The treatments based on antagonists targeting extracellular HMGB1 have shown encouraging results in several experimental models of infections and inflammation (Table 3). The current antagonists of HMGB1 that are studied most are ethyl pyruvate (EP), glycyrrhizin (GL), diflunisal (DLF), triptolide, and other peptides [33]. EP is an ester derivate of pyruvic acid, an end-product of glycolysis. EP is considered a protective antioxidant that limits ROS damage during inflammatory disease [122]. In animal models, EP reduces the lethality of hemorrhage shock and correlates with HMGB1 reduction in mice’s serum [123].

EP also suppresses RAGE expression, contributing to the attenuation of mesothelioma malignancy [124]. Moreover, EP inhibited NSCLC cell growth, invasion, and migration and induced apoptosis by suppressing the HMGB1/RAGE axis [125].

Triptolide is a natural diterpenoid triepoxide derived from the traditional Chinese herb Tripterygium Wilford [126]. Triptolide suppresses the expression and release of HMGB1 in breast cancer cells in vitro and in vivo. Triptolide has a synergistic effect with EP in the inhibition of cancer cell proliferation; this effect is mediated by the HMGB1/TLR4/NF-κB axis [127].

DFL is a salicylic acid derivate with anti-inflammatory activities, which has been approved by the FDA for treatment of chronic inflammatory diseases. DFL inhibits HMGB1 by binding to the HMGB1/CXCL12 heterocomplex and preventing the recruitment of inflammatory cells [128].

GL is a natural compound extracted from the licorice plant, *Glycyrrhiza glabra*, that directly binds HMGB1, thereby inhibiting it. GL decreases HMGB1 cytoplasmic transduction and suppresses the expression of inflammatory cytokines [129]. GL blocks the binding of extracellular HMGB1 with its receptors by directly binding to the A box and B box domains [130]. GL also mitigates radiation-induced acute lung damage by inhibiting the HMGB1/TLR4 pathway [131].

Recently, P5779, a tetramer peptide that acts as an inhibitor of HMGB1, targeting HMGB1/TLR4/MD-2 pathway, has been created. In human macrophages, P5779 mimetic peptides inhibited TNF-α release induced by HMGB1. P5779 can also ameliorate inflammatory disorders driven by HMGB1/TLR4 [132].

HMGB1 secretion can be inhibited by other small molecules such as metformin [133,134], vitamin D [135], and nuclear factor-erythroid 2-related factor 2 (Nrf2)/hemeoxygenase-1 (HO-1). In vitro studies indicated that metformin, a first-line antidiabetic drug also known for its anti-inflammatory effects, decreases HMGB1 expression, inhibits its translocation to cytosol, and protects against hyperglycemia-induced cardiomyocyte injury by inhibiting RAGE [134].

It was reported that the Nrf2/HO-1 pathway suppresses HMGB1 translocation and secretion. In macrophages, lipopolysaccharide (LPS)-induced HMGB1 secretion can be inhibited by anti-inflammatory vitamin D through the Nrf2/HO-1 pathway [136,137].

In recent years, several biological compounds have been developed to modulate HMGB1, including polyclonal and monoclonal antibodies and soluble receptors. The soluble RAGE (sRAGE), an isoform of RAGE, is present in the bloodstream and in the extracellular matrix (ECM). It can be considered a biomarker for HMGB1 related diseases, such as cancer. The sRAGE acts as a decoy to compete with RAGE in ligand-binding and has been used to block the HMGB1/RAGE pathway in tumors of animal models. Soluble RAGE can reduce HMGB1 concentration in ECM and decrease the stimulatory effects of HMGB1 on cancer cell proliferation and migration [33,120]. Polyclonal and monoclonal antibodies against HMGB1 antagonize extracellular HMGB1 in many experimental models. Some reports indicate that polyclonal antibodies against HMGB1 are studied on animal models but are not appropriate for clinical applications. In a mouse model of hemorrhagic shock, HMGB1 was released during shock following trauma damage. The polyclonal antibody against HGMB1 has been used to increase survival and limit gut barrier dysfunction [138]. HMGB1 is involved in hepatic ischemia and reperfusion; in a mouse model of liver injury, the treatment with neutralizing antibodies against HMGB1 was protective in ischemia/reperfusion events [139]. Intraperitoneal injection of anti-HMGB1 antibodies in a mouse model of bleomycin-induced pulmonary fibrosis reduced lung inflammation and fibrosis [140]. In order to overcome the limits of the clinical application of polyclonal antibodies, monoclonal antibodies (mAb) have been developed. Anti-HMGB1 mAb therapy is beneficial for the treatment of different inflammatory diseases using animal models [141]. The use of monoclonal antibody 2G7, which binds to an epitope of HMGB1 in the A box, shows beneficial therapeutic effects in experimental models of sepsis [142]. DPH1.1 mab against the 17-merpeptide P1 at the end of the B box blocks HMGB1 caused the cell migration of 3T3 fibroblasts in vitro and leucocyte recruitment in vivo. Furthermore, intravenous injection of anti-HMGB1 mAb protects against BBB disruption induced by ischemia and hemorrhage in rats. The positive effects of treatment with HMGB1 mab have been proven in several preclinical models, but further clinical studies are needed [82,143]. The blockade of HMGB1 with monoclonal antibodies may represent a new direction in the suppression of several inflammatory conditions [144].

As described above, the role of epigenetic regulation on HMGB1 and cancer is intriguing. Many miRNAs have been described as HMGB1 modulators that can act alone or in combination with chemotherapy drugs to reduce tumor growth. The HMGB1 gene contains response elements in its 3′-untranslated (3′ UTR) regions targeted by miRNAs. In cervical cancer, upregulation of miR-34a, miR-142, and miR-1284 suppress HMGB1 mRNA levels, reducing cancer cell proliferation, migration, and invasion [145]. Moreover, miR-21, miR-129-2, miR-200a, miR-320a, miR-325, and miR-505 are involved in dysregulation of HMGB1 signaling in human HCC. Their expression levels are inversely correlated with HMGB1 [54]. Overall, the modulation of the miRNA/HMGB1 axis may provide new therapeutic strategies for cancer.

The crosstalk between miRNAs and emerging noncoding RNAs can modulate HMGB1 expression. In a model of inflammation and kidney tubular cell injury caused by intrarenal calcium oxalate crystals, the interaction between the long noncoding RNA, H19, and miR-216b inhibited HMGB1 expression by directly binding its 3′-UTR, blocking the HMGB1/TLR4/NF-κB pathway [146]. In addition, the interaction between circular RNAs and miRNAs can modulate the expression of HMGB1. A recent study shows that circRNA-101368, which target miR-200a, are upregulated in hepatocarcinoma (HCC) tissue samples. This overexpression correlates with poorer prognosis for patients. MiR-200a has been negatively correlated with circRNA-101368 and HMGB1, while circRNA-101368 and HMGB1 was positively linked. CircRNA-101368 knockdown suppressed shuttle and protein expression of HMGB1, RAGE, and NF-κB, while it increased E-Cadherin expression in HCC cells. These data demonstrate a network of circRNAs-miRNA-mRNA in HCC and provide a novel mechanism of HCC cell migration regulation [147].

Although some HMGB1 therapeutic compounds are potentially eligible for clinical trials in HMGB1-driven inflammatory diseases, clinical studies are still ongoing. Nowadays, there are 104 proposed clinical trials on HMGB1 as a biomarker or therapeutic target in different pathologies. Among these trials, 10 have been completed in cancer, 3 in respiratory diseases, 3 in neurological disorders, 1 in diabetes, and 5 in cardiovascular diseases (www.clinicaltrials.gov, accessed on 15 October 2021). The data indicate the impressive interest in HMGB1 as a new biomarker and therapeutic target of the most common pathologies in the world.

## 4. Conclusions, Remarks, and Future Perspectives

The ability to self-repair is crucial to evolutionary success. A new field of medicine, regenerative medicine, focuses on understanding and exploiting the body’s resources to limit the damage caused by pathogens, trauma, and even degenerative diseases, to reach or improve recovery.

Whereas molecules or parts of molecules or polymeric complexes common to entire classes of pathogens are no self and alert the body to microbial invasion, endogenous (self) molecules can also signal danger and traumatic cell death or distress. HMGB1 is a danger signal that originates from damaged self rather than pathogens.

HMGB1 proteins are extremely conserved in evolution, indicating comparable biological functions in human and other animal models. All mammals have 3 different genes, Hmgb1, 2, and 3, with coding for HMGB proteins that share about 80% identity and differ for the length of their acidic tail. In mice, for example, the Hmgb1 gene is essential; new-born mice with mutations in the Hmgb1 gene die within a few hours due to their inability to use glycogen stored in the liver. Moreover, these mice develop several defects if nurtured in the first days of life with parenteral glucose delivery. Conversely, mutated mice lacking Hmgb 2 or 3 genes have few defects. Inasmuch as HMGB1 is a prototypical alarmin evolutionarily conserved, our literature analysis indicates that the studies conducted on animal models on HMGB1 and cancer, diabetes, cardiovascular, respiratory, and neurological diseases have a prevalence comparable to reports on HMGB1 in human subjects.

Numerous in vivo and in vitro studies have shown HMGB1′s therapeutic potential. In animals, intravenous injections of HMGB1 exert a beneficial effect on myocardial ischemia–reperfusion injury, mediating activation of the inflammatory-reparative response. Several reports demonstrate that, in pathological conditions, targeting HMGB1 reduces tissue damage caused by inflammation. HMGB1 blockaded with monoclonal antibodies may represent a new direction in the management of inflammatory chronic conditions. In addition, endogenous HMGB1 can be useful as a diagnostic and prognostic biomarker in human cardiovascular disorders. The extracellular HMGB1 blockade can also induce beneficial effects in cancer management. Recently, it was reported that targeting extracellular HMGB1 causes a deep remodeling of the tumor-immune microenvironment, improving the efficacy of anti-PD-1 cancer mono-immunotherapy. Extracellular HMGB1 blockade may complement first-generation cancer immunotherapies by remobilizing the antitumor immune response. Despite these results, some questions need to be resolved. The exact mechanism of HMGB1 translocation from the nucleus to cytoplasm and its release in extracellular space is not fully understood. Since HMGB1′s different redox status induces in situ modulations, it would be interesting to clarify the specific functions of each HMGB1 isoform. Moreover, multiple HMGB1 receptors with various degrees of cell preference add another layer of complexity in studying HMGB1′s role and its antagonists in different diseases. Despite these difficulties, targeting HMGB1 has been proven successful in the treatment of inflammation and inflammatory diseases, especially in sepsis, inflammation, autoimmune diseases, and cancer. Blocking excessive amounts of extracellular HMGB1, principally the disulfide isoform is an attractive clinical prospective to ameliorate systemic inflammatory diseases. Further efforts in the study of HMGB1 in the most socially relevant pathologies can help to fill the gaps in our knowledge and bring HMGB1 closer to the next step of therapy: the use of HMGB1 as druggable molecules.

## Figures and Tables

**Table 1:** Number of Records Published Yearly between 2006 and 2021 per Disease and Category (Studies on Human Subjects).

	2006	2007	2008	2009	2010	2011	2012	2013	2014	2015	2016	2017	2018	2019	2020	2021	TOTAL
Acute Myocardial Infarction	0	1	1	2	0	0	1	2	1	5	2	2	3	2	3	1	**26**
Chronic Heart Failure	2	2	1	1	6	7	7	10	5	8	9	7	16	6	11	2	**100**
Peripheral arterial disease	0	0	0	0	1	0	0	1	1	2	0	3	2	1	0	0	**11**
**TOTAL CARDIOVASCULAR**	**2**	**3**	**2**	**3**	**7**	**7**	**8**	**13**	**7**	**15**	**11**	**12**	**21**	**9**	**14**	**3**	**137**
**Cancer**	**10**	**26**	**26**	**35**	**37**	**40**	**71**	**77**	**90**	**104**	**89**	**95**	**126**	**133**	**110**	**26**	**1095**
Seizures	0	0	0	0	3	5	2	3	2	2	3	5	8	4	3	0	**40**
Parkinson’s Disease	0	0	0	0	0	0	1	4	1	0	2	0	4	3	2	0	**17**
Dementia	0	0	0	0	0	0	0	0	1	2	1	1	2	4	3	0	**14**
**TOTAL NEUROLOGICAL**	**0**	**0**	**0**	**0**	**3**	**5**	**3**	**7**	**4**	**4**	**6**	**6**	**14**	**11**	**8**	**0**	**71**
COPD	0	0	0	1	1	4	3	3	5	6	2	3	2	3	2	0	**35**
Asthma	0	1	0	1	2	6	5	4	3	10	5	7	4	9	10	0	**67**
Pulmonary hypertension	0	0	0	0	0	0	1	2	5	4	3	2	0	5	2	0	**24**
**TOTAL RESPIRATORY**	**0**	**1**	**0**	**2**	**3**	**10**	**9**	**9**	**13**	**20**	**10**	**12**	**6**	**17**	**14**	**0**	**126**
**Diabetes**	**3**	**2**	**4**	**12**	**13**	**15**	**19**	**22**	**19**	**17**	**25**	**18**	**17**	**23**	**13**	**3**	**225**

**Table 2:** Number of Records Published Yearly between 2006 and 2021 per Disease and Category (Studies on Animal Models).

	2006	2007	2008	2009	2010	2011	2012	2013	2014	2015	2016	2017	2018	2019	2020	2021	TOTAL
Acute Myocardial Infarction	0	0	0	0	0	0	0	0	0	0	0	0	1	0	2	0	**3**
Chronic Heart Failure	0	4	4	3	11	7	15	18	10	16	15	9	23	13	13	4	**165**
Peripheral arterial disease	0	0	0	0	1	0	0	0	0	0	0	3	2	0	0	0	**6**
**TOTAL CARDIOVASCULAR**	**0**	**4**	**4**	**3**	**12**	**7**	**15**	**18**	**10**	**16**	**15**	**12**	**26**	**13**	**15**	**4**	**174**
**Cancer**	**7**	**14**	**15**	**27**	**22**	**22**	**37**	**48**	**51**	**53**	**48**	**64**	**80**	**86**	**83**	**16**	**673**
Seizures	0	0	0	0	3	2	2	3	4	3	2	10	6	7	5	0	**47**
Parkinson’s Disease	0	0	0	0	0	0	0	0	0	1	0	1	2	2	4	0	**10**
Dementia	0	0	0	0	0	0	0	0	1	2	1	2	2	6	5	1	**20**
**TOTAL NEUROLOGICAL**	**0**	**0**	**0**	**0**	**3**	**2**	**2**	**3**	**5**	**6**	**3**	**13**	**10**	**15**	**14**	**1**	**77**
COPD	0	0	0	0	0	0	1	2	1	2	0	3	1	2	1	1	**14**
Asthma	0	0	1	0	2	0	3	2	9	10	5	12	8	9	12	1	**74**
Pulmonary hypertension	0	0	0	0	1	0	0	1	2	0	1	2	3	7	2	0	**19**
**TOTAL RESPIRATORY**	**0**	**0**	**1**	**0**	**3**	**0**	**4**	**5**	**12**	**12**	**6**	**17**	**12**	**18**	**15**	**2**	**107**
**Diabetes**	**3**	**2**	**5**	**9**	**15**	**15**	**15**	**19**	**26**	**17**	**33**	**32**	**23**	**23**	**19**	**7**	**263**

**Table 3:** HMGB1 Small-Molecule and Peptide-Based Antagonists (Not Comprehensive).

Inhibitor	In Vivo/In Vitro Effects	Pathway	Ref.
Ethyl pyruvate	HMGB1 reduction in mice’s serum, reduction of haemorrhage shock lethality, attenuation of mesothelioma malignancy (in vivo), inhibition of NSCLC cell growth (in vitro)	HMGB1/RAGE	[123,124,125]
Triptolide	Suppression of expression and release of HMGB1 in breast cancer cells (in vivo, in vitro)	HMGB1/TLR4/NF-κB	[126,127]
Diflunisal	Recruitment of inflammatory cells prevention (in vivo)	HMGB1/CXCL12	[128]
Glycyrrhiza	Decrease of HMGB1 cytoplasmic transduction (in vitro), mitigation of radiation-induced acute lung damage, neuroprotective effects (in vivo)	HMGB1/TLR4	[129,130,131]
P5779	Amelioration of inflammatory disorders (in vivo)	HMGB1/TLR4	[132]
Metformin	Decrease of HMGB1 expression, inhibition of its translocation to cytosol (in vitro), protection against hyperglycaemia-induced cardiomyocyte injury (in vivo)	HMGB1/RAGE	[133,134]
Vitamin D	Improvement of survival rate in septic mice, reduction of inflammatory response in asthmatic mice (in vivo)	Nrf2/HO-1–HMGB1/TLR4/NF-κB	[135,136,137]
sRAGE	Competition with RAGE in ligand-binding, decreasing stimulatory effects of HMGB1 on cancer cell proliferation and migration (in vitro)	HMGB1/RAGE	[33,120]

## Abbreviations

DAMPs: Damage-associated molecular pattern molecules; HMGB1: High-mobility group box 1; TLR; Toll-like receptors; RAGE: Receptor for advanced glycation end products; sRAGE: Soluble receptor for advanced glycation end products; NF-κB: Nuclear factor kappa-light-chain-enhancer of activated B; TNF-α: Tumor necrosis factor; IL: Interleukin; ROS: Reactive oxygen species; CXCL-12: C-X-C motif chemokine 12; CXCR-4: Chemokine C-X-C receptor 4; LPS: Lipopolysaccharide; mAb: Monoclonal antibodies.
